# Analysing hospital travel times in central Athens using Google Maps services: Nikolaos Christos Xifaras

**DOI:** 10.1093/eurpub/ckac130.119

**Published:** 2022-10-25

**Authors:** NC Xifaras, E Roubou, P Altsitzioglou

**Affiliations:** 1 Department of Medicine, National and Kapodistrian University of Athens, Athens, Greece; 2 Department of Orthopedics, Attikon University Hospital, Athens, Greece

## Abstract

**Introduction:**

The importance of timely care is well documented for numerous emergency conditions, including STEMI and ischemic stroke, where low symptom-to-balloon/symptom-to-needle times are crucial for mortality and disability. The study of all potential delays helps us understand the constraints we have to work under. Here, we use Google Maps services to map the travel times from central Athens areas to on-duty hospitals

**Methods:**

We built our code in the Python programming language, using the Google Maps Distance Matrix API to perform real-time trip duration calculations based on real-life data. As reference points, we used a set of Athens neighbourhoods provided by the Municipality as open data; we considered only public secondary and tertiary health facilities as valid destinations, and based our calculations based on the available daily duty schedules.

**Results:**

Our algorithm collected 43,200 data points in total over two weeks, using 144 starting points. The average trip durations to reach an on-duty department formed a right-skewed distribution (-0.424), with a mean of 19.55 minutes and a median of 19.95 minutes. The maximum average time was 26.78 minutes, and the overall maximum was 44 minutes. Average travel times to cardiology, general surgery, neurology and internal medicine ERs, which experience a heavy patient load, were higher than the total mean (20.60/22.06/21.31/20.51 mins respectively). We found no correlation between the average travel time and average distance from a hospital or the geographical location, but we were able to create a map with hotspots of high/low travel times.

**Conclusions:**

Our approach to collecting accurate trip data has shown the impact of time-of-day and location on trips to hospitals, even for patients within the same larger area. As acute care can be time-sensitive, similar wide-scale modelling could be used to create systemic solutions, e.g. data-guided spatial distribution of facilities or transportation.

**Key messages:**

- Public APIs can be used to gather useful data about the context around our health systems.
- In Athens, a difference in position can mean up to 100% longer travel times to a hospital.

